# Functional and Structural Brain Damage in Friedreich's Ataxia

**DOI:** 10.3389/fneur.2018.00747

**Published:** 2018-09-06

**Authors:** Marinela Vavla, Filippo Arrigoni, Andrea Nordio, Alberto De Luca, Silvia Pizzighello, Elisa Petacchi, Gabriella Paparella, Maria Grazia D'Angelo, Erika Brighina, Emanuela Russo, Marianna Fantin, Paola Colombo, Andrea Martinuzzi

**Affiliations:** ^1^Severe Developmental Disabilities Unit, Scientific Institute, IRCCS “Eugenio Medea”, Conegliano, Italy; ^2^Neuroimaging Lab, Scientific Institute IRCCS “Eugenio Medea”, Bosisio Parini, Italy; ^3^Department of Information Engineering, University of Padova, Padova, Italy; ^4^NeuroMuscular Unit, Department of NeuroRehabilitation, IRCCS “Eugenio Medea”, Bosisio Parini, Italy

**Keywords:** Friedreich's ataxia, neuroimaging, diffusion tensor imaging, fMRI, biomarkers, disease severity measures

## Abstract

Friedreich's ataxia (FRDA) is a rare hereditary neurodegenerative disorder caused by a GAA repeat expansion in the *FXN* gene. There is still no cure or quantitative biomarkers reliaby correlating with the progression rate and disease severity. Investigation of functional and structural alterations characterizing white (WM) and gray matter (GM) in FRDA are needed prerequisite to monitor progression and response to treatment. Here we report the results of a multimodal cross-sectional MRI study of FRDA including Voxel-Based Morphometry (VBM), diffusion-tensor imaging (DTI), functional MRI (fMRI), and a correlation analysis with clinical severity scores. Twenty-one early-onset FRDA patients and 18 age-matched healthy controls (HCs) were imaged at 3T. All patients underwent a complete cognitive and clinical assessment with ataxia scales. VBM analysis showed GM volume reduction in FRDA compared to HCs bilaterally in lobules V, VI, VIII (L>R), as well as in the crus of cerebellum, posterior lobe of the vermis, in the flocculi and in the left tonsil. Voxel-wise DTI analysis showed a diffuse fractional anisotropy reduction and mean, radial, axial (AD) diffusivity increase in both infratentorial and supratentorial WM. ROI-based analysis confirmed the results showing differences of the same DTI metrics in cortico-spinal-tracts, forceps major, corpus callosum, posterior thalamic radiations, cerebellar penduncles. Additionally, we observed increased AD in superior (SCP) and middle cerebellar peduncles. The WM findings correlated with age at onset (AAO), short-allelle GAA, and disease severity. The intragroup analysis of fMRI data from right-handed 14 FRDA and 15 HCs showed similar findings in both groups, including activation in M1, insula and superior cerebellar hemisphere (lobules V–VIII). Significant differences emerged only during the non-dominant hand movement, with HCs showing a stronger activation in the left superior cerebellar hemisphere compared to FRDA. Significant correlations were found between AAO and the fMRI activation in cerebellar anterior and posterior lobes, insula and temporal lobe. Our multimodal neuroimaging protocol suggests that MRI is a useful tool to document the extension of the neurological impairment in FRDA.

## Introduction

Friedreich's ataxia (FRDA) is an autosomal recessive progressive hereditary neurodegenerative disorder caused by a GAA repeat expansion in the first intron of the *FXN* gene on chromosome 9 (1). The prevalence in the Caucasian populations is 2–5:100.000 (2). FRDA is characterized by early onset and progressive deterioration of the motor and sensory functions, scoliosis, cardiomyopathy, and eventually nystagmus (3, 4). Age at onset (AAO), clinical progression and severity are not uniform across patients, but variably correlate with the short-allele expansion size (5). Magnetic Resonance Imaging (MRI) studies have provided several insights over the damage in cerebellar, cerebral, and spinal cord areas involved in FRDA.

Cerebral, cerebellar, and spinal cord involvement in FRDA has been documented and established with different MRI-based techniques. Volumetric MRI studies have shown widespread involvement of white (WM) and gray matter (GM). Atrophy has been documented in the infratentorial compartment at the level of dentate nuclei (DN) (6), peridentate WM (7), posterior cerebellar lobules, vermian cortex, as well as in the dorsal medulla (7–10) and in supratentorial GM areas (6, 8, 9). Diffusion Tensor Imaging (DTI) studies further characterized structural changes in WM revealing alterations in the cerebellar peduncles (6, 11–16) and in the cerebellum (11, 13). Alterations in the corticospinal tracts (CST) were observed at the level of subcortical pre-central WM, posterior limb of internal capsule (PLIC) (11) and in the brainstem (6, 11, 13, 15). Furthermore, other DTI based studies have reported alterations of the posterior thalamic radiations (14, 15, 17), optic radiations (18), and the long associative tracts (11, 14, 15).

Few functional MRI (fMRI) studies have been performed in FRDA, including either motor (19–23) or non-motor tasks (15, 24, 25). These studies reported overall significant differences of activation in the posterior cerebellar lobules (20, 22) and in the cortical motor and sensory areas (19–22).

The rationale behind the present work is the existence of a pattern of functional and structural alterations characterizing WM and GM in FRDA which correlates with specific clinical measures known as ataxia scales (26–28) routinely used to assess disease severity. Once identified, this pattern could be directly used for the implementation of longitudinal studies possibly overcoming some of the sensibility limitations recently demonstrated for the clinical scales (29, 30). For this reason, we designed a cross-sectional study of FRDA from a neuroimaging (Voxel-Based Morphometry, DTI, fMRI) and a clinical prospective in order to provide a composite overview of the CNS damage in FRDA versus healthy controls (HCs). In addition, we investigated the correlation between clinical functional scales and neuroimaging metrics.

## Materials and methods

### Participants

We recruited a cohort of 21 patients with a molecularly confirmed diagnosis of FRDA at the research centers “Eugenio Medea” in Conegliano/Pieve di Soligo (TV, Italy) and Bosisio Parini (LC, Italy). The recruited patients were older than 12 years and had an early onset FRDA (under 25 years old). All participants, but three, were native Italians, mostly originating from Central and North Italy. Non-Italian patients came from Albania (*n* = 2) and Germany (*n* = 1). A group of 18 age and sex matched HCs was recruited for inter-group comparison following a detailed anamnestic interview and cognitive assessment. The demographic data of patients and controls are presented in Table 1.

**Table 1:** Demographic and clinical data.

	**FRDA (*n* = 21)**	**HC (*n* = 18)**
	**Mean ±SD (range)**	**Mean ±SD (range)**
Gender F (%)	16 (76.19)	11 (61.11)
Hand dominance	19R, 2L	18R
AAV (y)	26.95 ± 10.35 (12–50)	27.05 ±9 (16–46)
GAAsr	671.24 ± 210.5 (170–946)	–
GAAlr	812.6 ± 225.04 (350–1230)	–
AAO (y)	10.62 ± 4.58 (4–20)	–
DD (y)	16.33 ± 8.82 (3–32)	–
SARA	21.38 ± 7.76 (8–32)	–
ICARS	52.95 18. 53 (22–84)	–
FARS -ne	62.25 ± 19.37 (31.33–92.5)	–

*FRDA, Friedreich's Ataxia, HC, healthy control GAAsr, GAA short repeat; GAAlr, GAA long repeat; SARA, Scale for the Assessment and Rating of Ataxia (0–40); ICARS, International Cooperative Ataxia Rating Scale (0–100); FARS-ne, Friedreich Ataxia Rating Scale -neurological examination (0–117)*.

### Ethic committee approval and patients consent

The study has been reviewed and approved by the Ethic Committee IRCCS E. Medea—Associazione La Nostra Famiglia—Bosisio Parini (LC) (Prot. No 051/11-CE) and all participants gave their written informed consent in accordance with the Declaration of Helsinki.

### Clinical measurement tools

All patients underwent a complete clinical and neurological assessment. The Scale for the Assessment and Rating of Ataxias scale (SARA) (28), International Cooperative Ataxia Rating Scale (ICARS) (26), and the neurological section of the Friedreich Ataxia Rating Scale (FARS) (27) were implemented.

Patients and HCs underwent a cognitive assessment specific for 2 age groups: 12–16 and 16–50 years old. The cognitive functions of the subjects aged 12–16 years were assessed by using the Wechsler Intelligence Scale for Children III (WISC-III) (31). The group of adults and older adolescents in both FRDA and HCs were assessed by using the Wechsler Adult Intelligence Scale Revised (WAIS- R) (32).

### Neuroimaging protocol

FRDA and HCs underwent an MRI session with a 3T Philips Achieva Scanner (Philips Medical System, The Netherlands), equipped with a digital 32-channel head coil. The acquisition protocol included a T1-weighted (T1w) high resolution sequence (TE/TR = 3.5/8 ms, flip angle 8°, SENSE factor 2, voxel-size 1 × 1 × 1 mm^3^ matrix size 256 × 256 × 160), a multi-shell diffusion MRI acquisition (15 directions at b = 300 s/mm^2^, 53 directions at b = 1,100 s/mm^2^, 8 volumes at b = 0 s/mm^2^, TE/TR = 100/8,800 ms, SENSE factor 2, SPIR fat suppression, voxel size 2.2 × 2.2 × 2.2 mm^3^, matrix size 112 × 112 × 80), a T2-weighted (T2w) fat suppressed scan (for DTI processing purposes, TE/TR = 100/4,700 ms, SENSE factor 2, voxel-size 1.5 × 1.5 × 1.5 mm^3^, matrix size 160 × 146 × 110), and a fMRI sequence (FOV = 240 × 240 mm^2^, 40 slices interleaved without gap, voxel size 2.5 × 2.5 × 3.5 mm^3^, TE/TR = 20/2,000 ms, flip angle 85°, 178 time points).

### fMRI motor task

The fMRI protocol included a standard block design finger tapping task involving both hands. Subjects were asked to press the buttons of an MRI-compatible response-device using all the fingers in sequence from the thumb to the little finger, always starting from the thumb. Blocks lasted 20 s for each hand with 16 s inter-stimulus interval. The fMRI task was paced according to the screen commands that were provided with a regular pattern. A drawing of the right or left hand with a caption (“right hand” or “left hand”) was projected on MR compatible goggles worn by the patients during the stimulus. A fixation point was projected in the inter-stimulus interval. Subjects were trained before the scan to familiarize with the projected instructions and with the hand device and to ensure comprehension of the task.

### Gray matter analysis

Voxel-Based Morphometry (VBM) pipeline (33) was applied to T1w images to detect morphological differences in the volumes between patients compared to HCs. Data was pre-processed with the N4 tool of ANTs (34, 35) to remove intensity field inhomogeneity, then the FSL anatomical pipeline was applied (36–38) to remove the skull and perform tissue segmentation, finally obtaining the Partial Volume Estimate (PVE) map of GM. A population template of GM was built among all subjects (including patients) with ANTs (34, 39, 40), then the GM PVE of each subject was non-linearly moved to template space and multiplied by the determinant of the transformation Jacobian. Voxel-wise statistics were computed with a general linear model (GLM) using FSL (41), with age, sex, and intracranial volume (ICV) in native space as covariates. The critical value for null hypothesis rejection was set at 0.05 corrected for multiple comparisons and employing the TFCE technique (42).

### White matter analysis

All the individual DTI data werepre-processed with Tortoise (43), taking advantage of the T2w acquisition to correct for motion and eddy current artifacts. With the same software the mono-exponential non-linear model was fitted on the data including all shells (44, 45) to compute the diffusion tensor and DTI derived measures to associated quantitative metrics, as fractional anisotropy (FA), axial diffusivity (AD), mean diffusivity (MD), and radial diffusivity (RD). Two study specific templates were built with DTI-TK (46, 47), one for HCs and one for patients. DTI-TK takes advantage of all the tensor elements to perform the registrations, delivering more accurate spatial alignments than tools based on intensity registration, especially in regions with complex fiber architecture such as the brainstem. The template of FRDA was non-linearly registered to the template of HCs, therefore then the transformations were concatenated to move the quantitative maps of FRDA subjects to the template space of HCs with a single interpolation. Statistical analysis were performed both at region of interest (ROI) and at voxel level. Voxel-wise statistics were performed for each DTI derived map with a general linear model (GLM) using age and sex as covariates, with the same approach previously described for VBM. To perform ROI level statistics, we moved the two JHU DTI-based WM atlas (48) to the common space. For each of the 43 regions defined in the atlas (see list in Table S1), we tested FA, MD, AD, and RD with a GLM comparing HCs and FRDA patients while accounting for age and sex as covariates. For the ROI level analysis, the critical threshold was set to 0.05 corrected with the Bonferroni method for multiple comparisons.

### Functional MRI analysis

Data were pre-processed using SPM12 (49) and ANTs. Functional volumes were realigned using a two-step realignment process (to the first volume of the sequence and then to the mean volume), then the mean volume was co-registered to the T2w anatomical volume to correct for EPI distortions (50). The T2w volume was rigidly aligned to the corresponding T1w anatomical volume after normalization to the MNI152 space. Finally, all the transformations were combined together to avoid further interpolation errors. The combined transformation was applied to normalize the functional sequence on the MNI space, with a final voxel size of 2 × 2 × 2 mm^3^. Before the single-subject analysis, a Gaussian spatial filter, with FWHM equal to 6 mm, was applied to the functional data to increase the Signal-to-Noise-Ratio (SNR) and to deal with the residual anatomical differences between subjects. Additionally, a high-pass temporal filter with cut-off frequency of 128 s was used to correct for slow signal drifts. The GLM approach was adopted for the single-subject analysis: the design matrix included one regressor for each condition of interest: rest, right (R) hand movement, left (L) hand movement and one regressor of no interest for each as nuance factors (movement parameters of the realignment process and outlier volumes). The ARtifact Detection Tool (ART) was employed to create regressors and detect outliers (51). A volume was defined as an outlier when its displacement from the reference volume was >2 mm or when its global mean Z-score was >5. After the estimation of the model was completed, we created a map for each effect we wanted to test. Contrast maps between the R and the L hand movement task (R>L and L>R) were computed to exclude confounding factors due to the non-motor component of the task (attention, visual stimulation). These maps were subsequently used as inputs for the following GLM group analysis. A one-sample *t-*test was performed among the two groups individually to verify which anatomical regions were involved in the task, then a two-sample *t-*test was performed to verify differences between groups. All the statistics were performed with SPM12 using a threshold *p* < 0.05 corrected for the False Discovery Rate (FDR) at voxel level.

Following the same rationale of statistical analysis performed on the DTI data, in the fMRI dataset we performed a ROI-based GLM correlation analysis of beta values, including the clinical variables as predictors. However, differently from the DTI dataset, the fMRI dataset included only 2 male subjects. For this reason, the sex of patients was not included as confounding factor. For the correlation analysis we used clusters of significant activations generated by R hand and L hand movement (contrasts “R Hand > L Hand” and “L Hand > R Hand) in the HC group. This approach guaranteed the independence of the ROI-selection step from the data used in the GLM correlation analysis. We found eight ROIs related to the movement of the R hand only, and seven ROIs related to the movement of the L hand only. Significance was set using a *p* < 0.05 corrected for the number of variables tested.

### Statistical analysis

Computed descriptive statistics mainly included mean values, modes and standard deviations of the variables. Inferential statistics on the clinical variables were performed with SPSS v24. Further statistical investigations were performed within the group of FRDA patients to assess the relation between diffusion metrics FA and MD and clinical covariates scoring disease severity. The considered covariates were the functional scales SARA, ICARS, and FARS, the short repeat triplet GAAsr and the age at onset (AAO). Thus, a battery of multi-variate GLMs was fit for each ROI, following a design scheme with intercept, age, sex, and one additional covariate at time. The significance *p*-value used to consider a covariate significant was set to 0.05 divided by the total number of considered covariates.

## Results

### Clinical results

Twenty-one patients with a molecularly defined diagnosis of FRDA were examined. The mean age at the time of the clinical assessment (AAV) was 26.95 ± 10.35 years (range 12–50, mode 26 years) (Table 1). The mean disease duration (DD) was 16.33 ± 8.82 years (range 3–32). The gender ration was F/M: 16/5. Patients declared an AAO of about 10.62 ± 4.58 (range 4–20), with a bimodal distribution (10 and 11 years).

Twenty of the patients' cohort were homozygous for the GAA repeat expansion, and one presented a 170 GAA repeat expansion on one allele and a nonsense point mutation on the other (52). The mean GAA repeat expansion in the short-allele (GAAsr) was 671.24 ± 210.5 (range 170–946), while the long allele (GAAlr) counted for 812.6 ± 225.04 (range 350–1,230). The Spearman test correlations showed that GAAsr correlated negatively with the AAO (*R*^2^ = −0.692, *p* = 0.001) and positively with clinical severity scores SARA (*R*^2^ = 0.714, *p* = 0.000^*^) and ICARS (*R*^2^ = 0.690, *p* = 0.001). The DD correlated positively to all the clinical scales SARA (*R*^2^ = 0.529, *p* = 0.014), ICARS (*R*^2^ = 0.582, *p* = 0.006), and FARS-ne (*R*^2^ = 0.767, *p* = 0.016).

The clinical features of the patients at onset, and the involvement of the CNS, sensory and other systems derived from the neurological examination, the ataxia scales and other complementary assessments are shown in Table S2.

### Neuroimaging findings

#### Gray matter analysis

**Figure 1:**
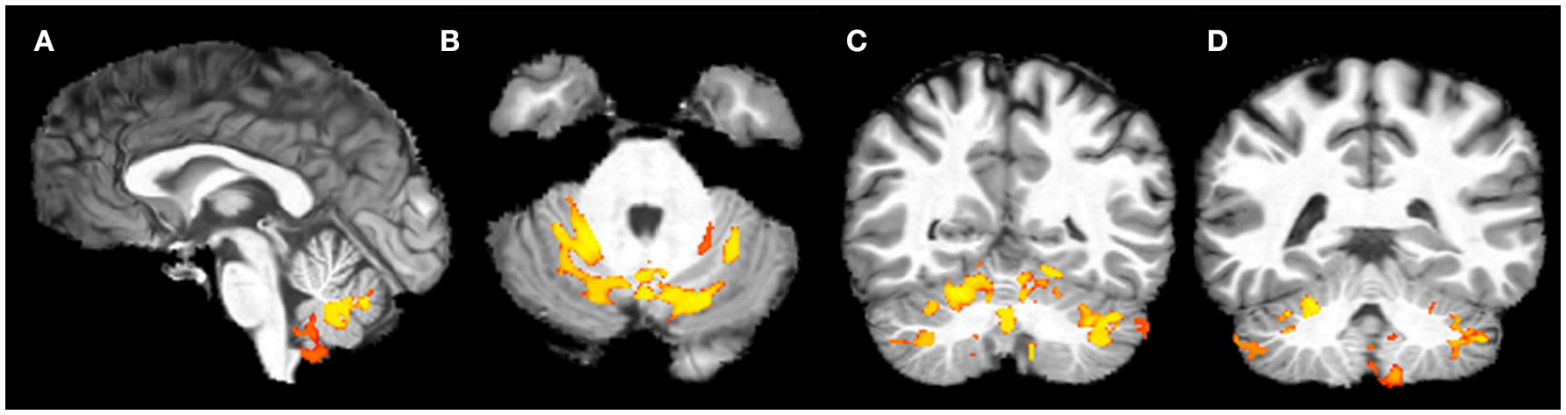
Voxel-based analysis of the Gray Matter reduction in FRDA patients compared to HCs. **(A)** Sagittal view, **(B)** Axial view, **(C,D)** Coronal view. FRDA, Freidreich's Ataxia; HCs, healthy controls. Results are overlaid on a subject from the cohort.

VBM showed a significant bilateral reduction of GM volume in lobule V, VI, VIII (L>R) and in the crus of cerebellum in FRDA patients compared to HCs. A significant volume reduction was observed also in the posterior lobe of the vermis, in both the flocculi and in the L tonsil (Figure 1). No differences between patients and controls were found in the supratentorial GM.

#### White matter analysis

**Figure 2:**
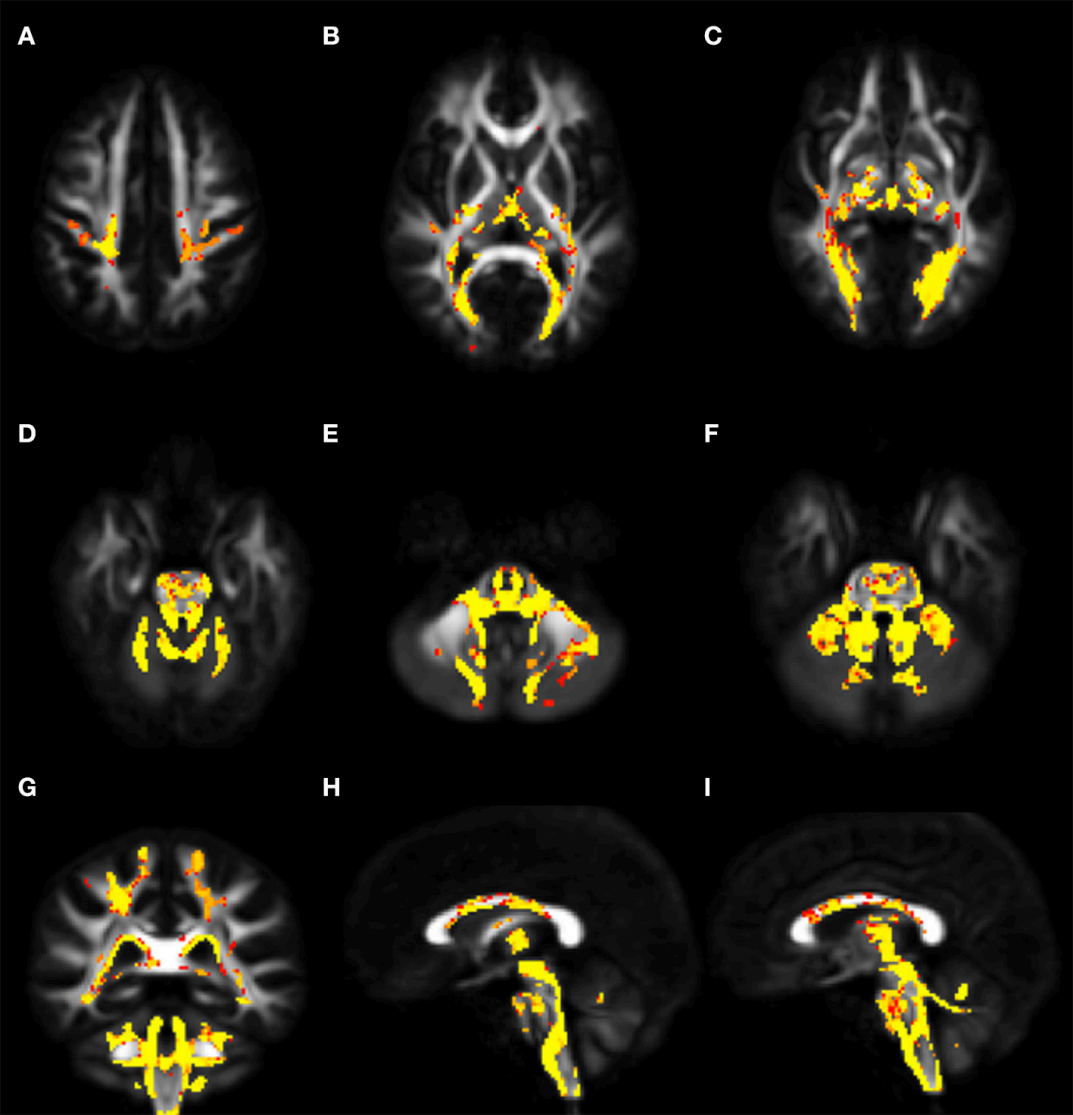
Voxel-wise analysis of White Matter in FRDA compared to HCs. The figure shows areas of FA reduction from the axial view **(A–F)**, coronal view **(G)**, and sagittal view **(H,I)**. FRDA, Freidreich's Ataxia; HCs, healthy controls; FA, fractional anisotropy.

**Figure 3:**
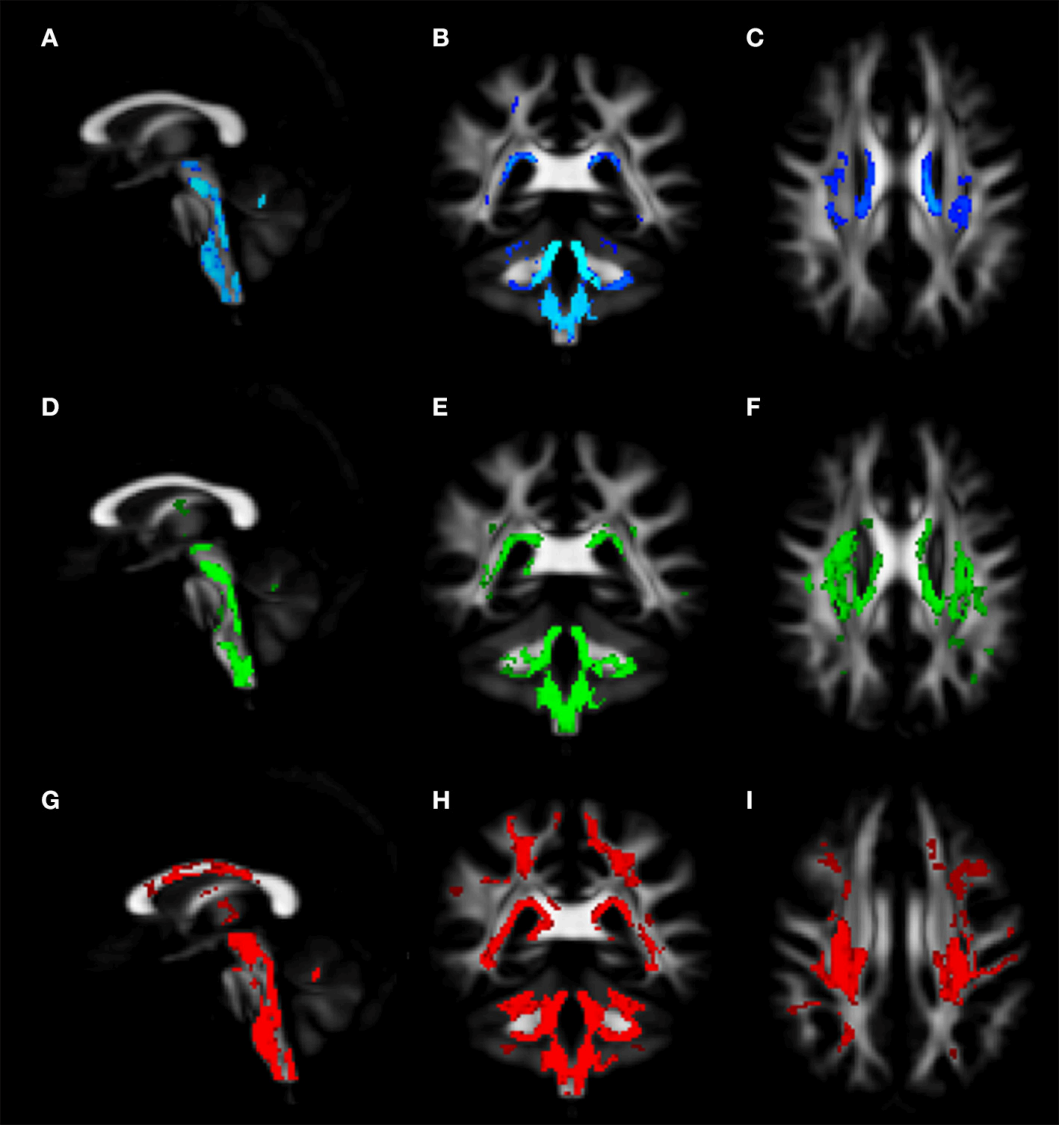
Voxel-wise analysis of White Matter in FRDA compared to HCs. The figure shows areas of MD increase **(A–C)**, AD increase **(D–F)**, and RD increase **(G–I)**. The distribution of the DTI indexes has been shown in the sagittal view **(A,D,G)**, coronal view **(B,E,H)**, and axial view **(C,F,I)**. FRDA, Freidreich's Ataxia; HCs, healthy controls; MD, mean diffusivity; AD, axial diffusivity; RD, radial diffusivity; DTI, Diffusion Tensor Imaging.

Voxel-wise analysis of DTI derived maps showed a diffuse reduction of FA values in both infratentorial and supratentorial WM of FRDA patients (Figure 2). In particular FA reduction involved deep cerebellar WM, inferior (ICP), middle (MCP), and superior (SCP) cerebellar peduncles (including the decussation in the brainstem), CST both in the brainstem, in the PLIC and in the subcortical WM close to motor cortex, posterior thalamic radiations and optic radiation, and corpus callosum (CC). Optic tracts were also partially involved. The same areas showed increased MD, AD, and RD values in FRDA patients, with a more diffuse pattern for RD (Figure 3).

The ROI-based analysis showed similar results. Significant FA reductions in FRDA patients were found in ROIs representing CSTs, forceps major, CC, posterior thalamic radiations, SCP, MCP, and ICP. The same areas showed increased MD and RD values, while AD was increased only in MCP and SCP (Table 2).

**Table 2:** DTI metrics distribution in the White Matter in FRDA and HCs and the intergroup variation.

**TRACTS**	**Mean FA [μm2/s]**			**Mean MD [μm2/s]**			**Mean AD [μm2/s]**			**Mean RD [μm2/s]**		
	***HC***	***FRDA***	***Variation %***	***HC***	***FRDA***	***Variation %***	***HC***	***FRDA***	***Variation %***	***HC***	***FRDA***	***Variation %***
SCP L	0.537	0.349	−34.9	3.065	4.400	43.6	1.667	1.958	17.4	0.699	1.221	74.7
SCP R	0.550	0.369	−33.0	2.984	4.099	37.4	1.646	1.865	13.3	0.669	1.117	66.9
ICP R	0.501	0.390	−22.2	2.164	2.607	20.5	.	.	.	0.505	0.682	35.0
ICP L	0.497	0.395	−20.5	2.190	2.605	19.0	.	.	.	0.514	0.679	32.2
Forceps major	0.506	0.447	−11.8	2.496	2.786	11.6	.	.	.	0.570	0.682	19.6
Body of CC	0.627	0.574	−8.6	.	.	.	.	.	.	0.447	0.552	23.6
Posterior thalamic radiation L	0.560	0.515	−8.0	2.209	2.407	9.0	.	.	.	0.480	0.557	16.1
CST R	0.510	0.467	−8.5	2.187	2.356	7.7	.	.	.	0.509	0.575	13.0
Posterior thalamic radiation R	0.561	0.517	−7.8	2.165	2.363	9.1	.	.	.	0.469	0.543	15.8
MCP and pontine crossing tract	0.490	0.453	−7.5	2.066	2.289	10.8	1.087	1.157	6.5	0.490	0.566	15.6
CST L	0.516	0.478	−7.4	.	.	.	.	.	.	0.496	0.551	11.2
Splenium of CC	0.654	0.614	−6.1	2.453	2.703	10.2	.	.	.	0.465	0.553	18.9

*GLM p < = 0.05 Bonferroni on FA. FA, Fractional anisotropy; MD: medial diffusivity; RD, radial diffusivity; HCs, healthy controls, FRDA, Friedreich's ataxia patients; SCP, superior cerebellar peduncle; L, left; R, right; ICP, inferior cerebellar peduncle; MCP medial cerebellar peduncle; CC, corpus callosum; CST, cortical spinal tract*.

No differences emerged at the level of association tracts like superior or inferior longitudinal fasciculi, uncinate fasciculi, or inferior fronto-occipital fasciculi.

The relative change of DTI-derived measures varied among significant tracts. The SCPs showed the most important alterations, with more than 30% FA reduction and more than 35, 13, and 65% increments in MD, AD, and RD values respectively (Table 2). The ICPs were the second most-affected tracts with a FA reduction around 20% and an increment of MD and RD of more than 18 and 32%, respectively.

Other significant tracts showed less severe modifications of DTI measurements.

### WM and clinical data correlation

Table 3 shows significant correlations between DTI-indexes (FA and MD) and clinical variables. Negative and positive correlations were found for FA and MD values. The AAO correlated with FA and MD in both supratentorial (thalamic radiations, CC, forceps, fornix, internal capsule) and infratentorial (ICP) tracts. Clinical scales and the number of GAA repetitions were negatively correlated with FA and positively correlated with MD in SCP, forceps, and fornix.

**Table 3:** Significant coefficients (β) of the GLM regression of DTI metrics with clinical covariates.

**Description**	**AAO(β)**	**GAAsr(β)**	**ICARS(β)**	**SARA(β)**	**FARS-ne (β)**
**FA**					
Anterior thalamic radiation L	0.0035	–	–	–	–
Anterior thalamic radiation R	0.0032	–	–	–	–
Posterior thalamic ratiation and retrolenticular part of IC R	0.0050	–	–	–	–
Posterior thalamic ratiation and retrolenticular part of IC L	0.0060	–	–	–	−0.0010
Forceps major	0.0059	−0.0001	−0.0013	−0.0029	–
Inferior fronto–occipital fasciculus R	0.0039	–	–	–	–
Body of CC	0.0071	–	–	–	–
Anterior limb of IC L	0.0049	–	–	–	–
Fornix (column, body, cres with stria terminalis)	0.0051	–	–	−0.0028	–
SCP R	–	−0.0001	−0.0016	−0.0040	−0.0016
SCP L	–	−0.0001	−0.0012	−0.0032	−0.0012
**MD**					
Anterior thalamic radiation L	−0.0283	–	–	–	–
Anterior thalamic radiation R	−0.0332	–	–	–	–
Posterior thalamic ratiation and retrolenticular part of IC L	−0.0347	–	–	–	–
CST R	−0.0280	–	–	–	–
Forceps major	−0.0360	–	–	0.0181	–
Inferior fronto–occipital fasciculus R	−0.0200	–	–	–	–
Superior longitudinal fasciculus L	−0.0229	–	–	–	–
Superior longitudinal fasciculus R	−0.0200	–	–	–	–
Splenium of CC	−0.0322	–	–	–	–
Fornix (column, body, cres with stria terminalis)	–	–	–	0.0220	0.0095
ICP R	−0.0322	–	–	–	–
SCP R	–	–	0.0137	0.0334	0.0132
SCP L	–	–	0.0120	0.0313	0.0119

*p < 0.05/nr of covariates. FA, Fractional anisotropy; MD, medial diffusivity; FRDA, Friedreich's ataxia patients; SCP, superior cerebellar peduncle; L, left; R, right; ICP, inferior cerebellar peduncle; CST, cortical spinal tract; AAO, age at onset; GAAsr, GAA short-allele; SARA, Scale for the Assessment and Rating of Ataxia (0–40); ICARS, International Cooperative Ataxia Rating Scale (0–100); FARS-ne, Friedreich Ataxia Rating Scale -neurological examination (0–117)*.

### fMRI analysis

Due to excessive motion artifacts and collaboration problems during the acquisition, 7 FRDA and 3 HCs were excluded from the following fMRI analysis. The FRDA group (*n* = 14) mean age was 27.6 ± 11.1 years (range 12.1–50.5) and gender ratio 12F/2M. The HCs (*n* = 15) group had a mean age 27.9 ± 9.8 years (range 15.9–45.7) and gender ratio 10F/5M. All the FRDA and HCs were R handed.

**Figure 4:**
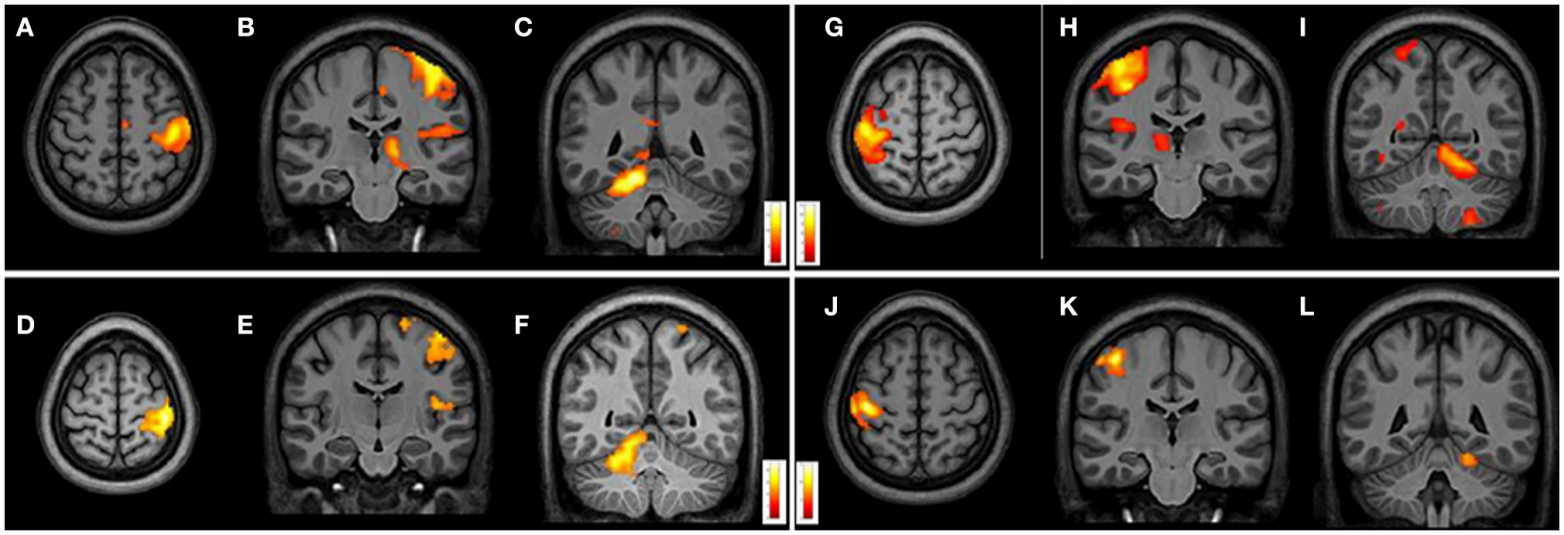
Cerebral and cerebellar fMRI intragroup analysis in FRDA and HCs showing similar areas of activation. Right-hand task **(A–F)** shows activated areas in transverse **(A,D)** and frontal views **(B,C,E,F)**. Left-hand task **(G–L)** shows activated areas in axial **(G,J)** and coronal views **(H–L)**. FRDA, Freidreich's Ataxia; HCs, healthy controls.

From intragroup analysis, in both FRDA and HCs, the multi-finger tapping task activated similar brain regions. In particular, while moving the R fingers (contrast R>L), significant activations were found in the L M1, L insula, and R superior cerebellar hemisphere (lobules V, VII, VIII). During the movement of L fingers (contrast L>R), activations involved R M1 cortex, R insula, and L superior cerebellar hemisphere (lobules V, VI, VIII) (Figure 4).

**Figure 5:**
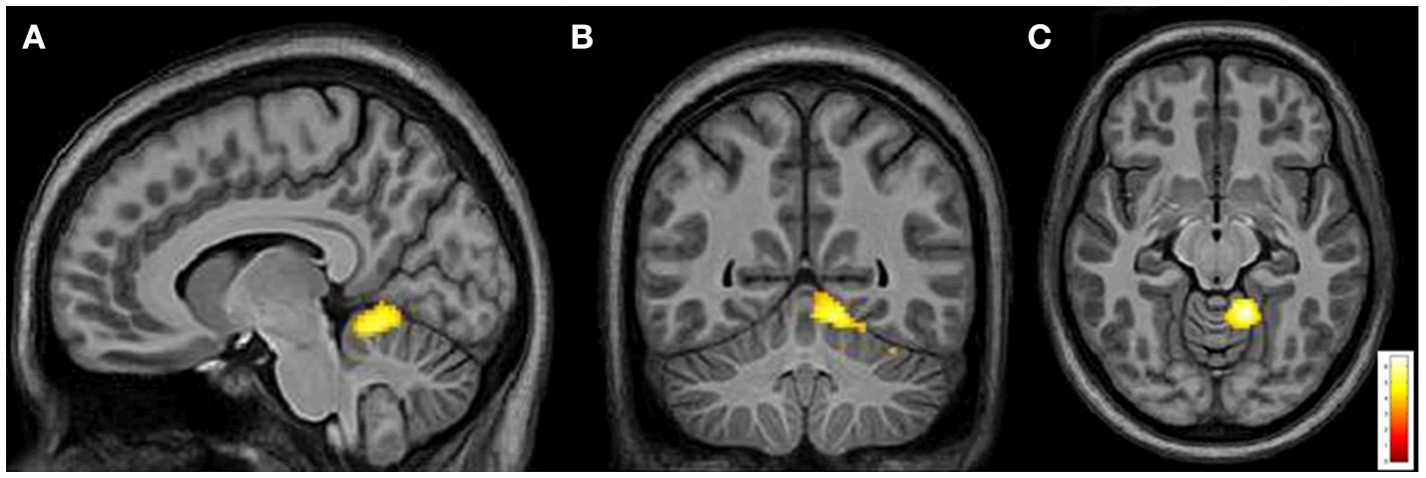
Cerebral and cerebellar fMRI intergroup analysis. Left-hand related activation differences between the FRDA and HCs. Higher activated areas are localized in the left superior cerebellar cortex: sagittal **(A)**, coronal **(B)**, and axial view **(C)**. FRDA, Freidreich's Ataxia; HCs, healthy controls.

From intergroup analysis, significant differences emerged only during the movement of the non-dominant hand (left-hand), with HCs showing a stronger activation (cluster size k = 42 voxels, *t* > 5.174, *p* < 0.05 FDR corrected) in the L superior cerebellar hemisphere (Figure 5). No significant differences were found during the movement of the dominant hand.

### fMRI and clinical data correlation

Table 4 shows the significant correlations between fMRI activation magnitude and the clinical parameters in the FRDA group. Significant positive correlations (*p* < 0.05/nr of variables) were found between AAO and activation in the cerebellar anterior lobe, insula, motor cortex, and temporal lobe. Negative correlations (*p* < 0.05) that did not survive to multiple comparisons correction, were found between disease severity measures (ICARS, SARA; FARS-ne), GAAsr and fMRI activation in the cerebellum, insula, and temporal lobes. Task-related cerebral activation generated more correlation during the R-hand task (dominant hand).

**Table 4:** Correlations between fMRI activation and clinical covariates.

	**AAO (β)**	**GAAsr (β)**	**ICARS (β)**	**SARA (β)**	**FARSne (β)**	**DD (β)**
**RIGHT-HAND TASK**						
Motor Cortex L	1.0139^*^	–	–	–	–	–
Middle temporal lobe	0.8804^*^	−0.622	−0.460	−0.465	−0.516	–
Insula L	1.0634^*^	−0.647	−0.671	−0.599	−0.620	–
Posterior Cingulum R	0.858	–	–	–	–	–
Cuneus R	0.904	–	–	–	–	–
Cerebellar anterior lobe (lobules V–VI) R	0.9486^*^	–	−0.602	–	–	–
**LEFT-HAND TASK**						
SMA R	–	–	–	–	–	−0.540
Cerebellar anterior lobe (lobules V–VI) R	0.9598^*^	−0.739	−0.648	−0.625	−0.587	–
Cerebellar posterior lobe (lobules VII–VIII) L	0.744	−0.758	−0.605	−0.647	−0.586	–

p < 0.05;

* *p < 0.05/nr of covariates. SMA, supplementary motor area; L, left; R, right; AAO, age at onset; DD, disease duration; GAAsr, GAA short-allele; SARA, Scale for the Assessment and Rating of Ataxia (0–40); ICARS, International Cooperative Ataxia Rating Scale (0–100); FARS-ne, Friedreich Ataxia Rating Scale -neurological examination (0–117)*.

### Cognitive assessment results

Nineteen patients underwent the cognitive assessment (Table S3). The protocol was not administered to two patients, as they were non-Italian speakers. The mean level of education was 12.84 years (SD ± 2.87). The intelligence quotient (IQ) level was normal in 13 FRDA (68.41%), borderline in 4 FRDA and in the intellectual disability range in 2 subjects (10.52%). In 4 out of 6 FRDA patients with reduced IQ, the verbal performance was more strongly impaired. In 10 cases, FRDA patients showed a disharmonic profile, with better verbal component. The IQ level components fell into the normal range in all the HCs.

Considering the IQ level as index of cognitive functioning and using an intellectual disability cut off of total IQ < 70; we found only 2 patients with cognitive deficits. Due to the unbalanced samples of patients with vs. without cognitive deficits, we did not perform group comparison of DTI and fMRI indexes. We tested the occurrence of any correlation between the IQ values and the DTI and fMRI metrics, but did not find any significant correlation.

## Discussion and conclusions

Our study demonstrates the extent of CNS brain damage in FRDA by using a composite protocol of clinical and multimodal neuroimaging tools as VBM, DTI and fMRI in a cross-sectional study. Our findings support the need for future longitudinal studies and highlights the possibility that MRI studies could provide valuable paraclinical biomarkers in FRDA.

### Voxel-based morphometry in the gray matter

By using the VBM technique we demonstrated infratentorial GM volume reduction, particularly in the lobules V, VI, VIII, crus of cerebellum, posterior lobe of vermis, flocculi bilaterally, and L tonsils. These results are consistent with previous studies (6, 8, 9, 15, 53) (Table S4). We did not find any supratentorial GM volume reduction or DN alteration. This is in line with reports of no cerebral atrophy in FRDA (11, 53), although opposite results have also been reported (6, 8, 15, 54).

Our findings support previous investigations reporting involvement of the anterior vermis and of the anterior cerebellar lobes (6, 11), otherwise known as the primary sensorimotor regions of the cerebellum.

Yet, we describe for the first time GM loss in FRDA in the lobule VIII, also known as the secondary sensorimotor cerebellum (55), in the flocculi bilaterally and in the left tonsil. This morphometric distribution of GM changes implies the involvement of both the cerebellar primary and secondary body maps in FRDA.

In addition, the GM reduction in the flocculi, in the lobules V–VI and in the dorsal vermis may provide the structural explanation for the eye movements impairments (Table S2) (56), as it was demonstrated in other neurodegenerative ataxias such as SCA6 (57) where the floccular atrophy was linked to gaze holding and pursuit impairment.

### Microstructural changes in the white matter

Voxel-wise and ROI-based analysis of DTI metrics reported diffuse microstructural changes in infra- and supratentorial WM. These findings are in line with previous DTI reports of alterations in the cerebellar peduncles (6, 11–16) (Table S4) and of the supratentorial involvement in areas such as CC, forceps major, posterior thalamic radiations, optic tracts, and optic radiations (6, 14, 15, 18, 58, 59). In particular, we observed significantly different FA and MD values between FRDA and HCs in the CST, as consistently reported in the literature (6, 11, 13, 15).

The intergroup variation of the four DTI metrics pointed toward a primary involvement of the SCPs and, to a less extent, of the ICPs. The infra- and supratentorial FA and MD values correlated with AAO in FRDA. In addition, the disease severity scored with SARA and FARS showed a strong correlation with FA and MD values in SCP and forceps major. These findings support previous correlating DTI metrics and disease severity scores in areas as the cerebellar peduncles and the CC (6, 7, 13, 16). Although other studies denied such correlations, we presume that this difference was due to the different techniques employed and study samples (12, 14, 59, 60).

We did not observe any structural alteration in the associative tracts. Others have reported FA reduction in the inferior fronto-occipital fasciculus (11, 14, 15) and in the inferior longitudinal fasciculus (11, 15). However, the FRDA intragroup FA and MD values correlated with the AAO in the inferior fronto-occipital and superior longitudinal fasciculi, suggesting sensitivity of this measure to damage over time.

The relevance of the consistent variation of the different DTI-indexes, in particular localized in the cerebellar peduncles and forceps major is strongly supported by the correlation with the different disease severity measures and with AAO.

### Motor function exploration

We observed similar areas of activation in both groups from the intragroup analysis of fMRI data with bi-manual finger tapping task. During the R-hand motor task (dominant hand in all the subjects) we observed significant activation in L M1, L insula, and the R superior cerebellar hemisphere (lobules V, VI, VIII). From the L-hand motor task, we observed significant activations of the R M1, R insula, and L superior cerebellar hemisphere (lobules V, VII, VIII). The areas activated in the cerebellum with R and L hand during the motor task were comparable to the areas found in other studies (20–23) (Table S4). Our intergroup analysis showed no significant differences during the dominant-hand task, differently from other studies that have reported either reduction of activation in M1, thalamus, DN, and lobule V (21, 22) or increase of activation in parietal cortex, striatum, supplementary motor area, and lobule VII (20–22) Interestingly, we found a significant difference during the non-dominant hand motor task with a stronger activation in the L superior cerebellar hemisphere in HCs when compared to FRDA. This difference was not previously described. One possible explanation is that while the dominant-hand is controlled by the dominant hemisphere, the non-dominant hand is controlled by both hemispheres (61, 62). The intergroup differences in our study might be explained by probable disruption in FRDA of the cerebellar circuitry involved in the primary somatosensory map in the cerebellum. In our study, we propose a bimanual device-mediated finger tapping task, while other studies have performed only dominant-hand motor tasks (19–22).

The correlation analysis demonstrates that the cerebral and cerebellar functionality can be related to clinical and genetic factors. Predominantly, the activation of the anterior cerebellum is directly correlated to AAO, GAAsr, and severity measures as reported elsewhere (4, 23).

Although the FRDA group had a lower IQ than HCs, the mean IQ level of both groups was within normal range, similarly to the results reported in Mantovan et al. (19). The lack of correlation between results of the neuropsychological assessment and either fMRI, VBM, or DTI data is not surprising since the finger tapping task was not constructed to explore cognitive relevant performances and the areas found impaired with VBM and DTI techniques are not primarily engaged in cognitive functions.

Our findings derive from a 3 Tesla scanner. This magnet strength was widely used in other studies (6, 8, 9, 15, 17, 20, 24, 25) (Table S4) and perhaps is sufficient to detect the CNS structural and functional damage in FRDA. Only two other works have tried a higher magnet field of 7 Tesla (22, 54) but their findings do not provide more insight.

Limitations in our study are the relatively small sample size, which impacts on the statistical strength of the study, the heterogeneity in disease severity of the recruited subjects and the lack of a longitudinal appraisal of both clinical and neuroimaging assessments. Few studies have recruited more patients, but the sample size was generally not remarkably different. The difficulty in recruitment of larger cohorts could be overcome by sharing common protocols in multiple centers and pooling the results. A step in this direction is already taking place with the ENIGMA network (*http://enigma.ini.usc.edu/*) which promotes neuroimaging data sharing for rare genetically determined diseases.

There have been few longitudinal studies in FRDA. Two longitudinal studies reported DTI changes as FA reduction in CC (6) and AD changes in CC splenium and WM deep subcortical parietal lobes after a follow-up period (59) with no macrostructural changes or atrophy. Two more longitudinal interventional studies have tried to investigate the effect of rhuEPO treatment in FRDA and showed increase in the GM volume in pulvinar and posterior parietal cortex (63) and WM bilateral increase in FA and increase in AD in cerebral hemispheres bilaterally (60). However, more longitudinal studies are needed in order to validate the cross-sectional study derived findings. This would lead to a better definition of the significance and of the temporal dynamics of the observed alterations. In such contexts, the neuroimaging study could provide the sensitive and objective biomarkers very much needed for the rapid and efficient clinical trial design and results evaluation.

In summary, we demonstrated infratentorial GM volume reduction suggesting an alternative sensorimotor cerebellar map involvement in FRDA with no supratentorial GM involvement. This finding shows the extension of cerebellar damage in FRDA.

The microstructural WM findings were consistent with the known areas of CNS damage and correlated with the clinical measures. The established correlation between DTI metrics and clinical variables as the AAO or the disease severity scores, should now be validated in larger cohorts and in longitudinal studies, which then could support the use of these measures as disease biomarkers in future studies. The non-dominant hand motor task differences between the FRDA and HCs gives hints on cerebral-cerebellar circuitry disruption in both hemispheres.

In conclusion, our multimodal imaging study provided convergent results, with a strong involvement of the cerebellar cortex, cerebellar WM tracts, in particular SCPs and ICPs and a strong functional involvement of the anterior lobe of the cerebellum during the non-dominant hand motor task. These findings bring a new dimensional role of the cortical circuitry involved in FRDA.

## Authors note

Paper previously presented by MV in part as a scientific poster at the 1st Congress of the European Academy of Neurology, June 20–23, 2015, Berlin, Germany.

The raw data supporting the conclusions of this manuscript will be made available by the authors, without undue reservation, to any qualified researcher.

## Author contributions

MV provided the design of the study, supervision, collected and analyzed the data, and prepared the manuscript. FA provided the design of the study and analyzed the data. AN and AD analyzed the data. SP provided the data and analyzed the data. EP, GP, MD, EB, ER, MF, and PC provided the data. AM provided the design of the study, supervision, and financial support. All authors revised the manuscript for important intellectual content, provided approval for publication and agree to be accountable for all aspects of the work.

### Conflict of interest statement

The authors declare that the research was conducted in the absence of any commercial or financial relationships that could be construed as a potential conflict of interest.
