# Supplementary material for: Functional and Structural Brain Damage in Friedreich's Ataxia
Source: Front Neurol. 2018 Sep 6;9:747. doi: 10.3389/fneur.2018.00747 (PMC6135889; doi:10.3389/fneur.2018.00747)
Supplement: Supplementary file 1 [file Table_1.pdf]

SUPPORTING INFORMATION

S1 TABLE. The 43 regions defined in the atlas for the DTI study. ROIs: regions of interest; SCP: superior cerebellar peduncle; L: left; R: right; ICP: inferior cerebellar peduncle; ICP: inferior cerebellar peduncle; CC: corpus callosum; IC: internal capsule.

**Considered ROIs**	
**Number**	**Region**
1	Anterior thalamic radiation L
2	Anterior thalamic radiation R
3	Cortico-spinal tract L
4	Cortico-spinal tract R
5	Cingulum (cingulategyrus) L
6	Cingulum (cingulategyrus) R
7	Cingulum (hippocampus) L
8	Cingulum (hippocampus) R
9	Forceps major
10	Forceps minor
11	Inferior fronto-occipital fasciculus L
12	Inferior fronto-occipital fasciculus R
13	Inferior longitudinal fasciculus L
14	Inferior longitudinal fasciculus R
15	Superior longitudinal fasciculus L
16	Superior longitudinal fasciculus R
17	Uncinate fasciculus L
18	Uncinate fasciculus R
19	Superior longitudinal fasciculus (temporal part) L
20	Superior longitudinal fasciculus (temporal part) R
21	Genu of CC
22	Body of CC
23	Splenium of CC
24	ICP R
25	ICP L
26	SCP R
27	SCP L
28	Anterior limb of IC R

29	Anterior limb of IC L
30	Posterior limb of IC R
31	Posterior limb of IC L
32	Anterior corona radiata R
33	Anterior corona radiata L
34	Superior corona radiata R
35	Superior corona radiata L
36	Posterior corona radiata R
37	Posterior corona radiata L
38	External capsule R
39	External capsule L
40	Middle cerebellar peduncle and pontine crossing tract
41	Fornix (column, body, cres with stria terminalis)
42	Posterior thalamic radiation and retrolenticular part of IC R
43	Posterior thalamic radiation and retrolenticular part of IC L
