# Supplementary material for: Functional and Structural Brain Damage in Friedreich's Ataxia
Source: Front Neurol. 2018 Sep 6;9:747. doi: 10.3389/fneur.2018.00747 (PMC6135889; doi:10.3389/fneur.2018.00747)
Supplement: Supplementary file 2 [file Table_2.pdf]

SUPPORTING INFORMATION

S2 TABLE. Clinical signs and symptoms in the FRDA cohort. Number of patients affected (percentages). FRDA: Friedreich's Ataxia; CNS: central nervous system.

**CNS involvement n (%)**		**Onset Symptoms n (%)**	
Dysarthria	18 (85.7)	Gait clumsiness,	14 (66.66)
Cerebellar signs	13 (61.9)	Cerebellar	9 (42.85)
Muscle weakness	21 (100)	Scoliosis	8 (38.1)
Muscle atonia	20 (95.2)		
Babinski positive	21 (100)		
Areflexia	21 (100)		
Nystagmus	13 (61.9)		
**Sensory Impairment n (%)**		**Other n (%)**	
Vibratory sense	21 (100)	Left ventricular hypertrophy	11 (52.38)
Touch	10 (47.6)	Dysphagia	17 (80.95)
Proprioception	7 (33.3)	Restrictive lung disease	3 (14.28)
Pain	5 (23.8)	Headache	3 (14.3)
Temperature	4 (19%)	Mood disorders	3 (14.3)
Hearing loss	3 (14.3)	Diabetes Mellitus	2 (9.5)
