# Supplementary material for: Functional and Structural Brain Damage in Friedreich's Ataxia
Source: Front Neurol. 2018 Sep 6;9:747. doi: 10.3389/fneur.2018.00747 (PMC6135889; doi:10.3389/fneur.2018.00747)
Supplement: Supplementary file 3 [file Table_3.pdf]

SUPPORTING INFORMATION

S3 TABLE. Intelligence quotient of the cohort. FRDA: Friedreich's Ataxia; IQ: intelligent quotient; SD: standard deviation.

**IQ**	**FRDA**		**HC**	
	mean	*SD*	*mean*	*SD*
IQ total	90,79	17,01	112,00	10,58
IQ verbal	99,47	16,93	118,00	9,33
IQ performance	83,84	20,18	101,78	13,51
