# Supplementary material for: Functional and Structural Brain Damage in Friedreich's Ataxia
Source: Front Neurol. 2018 Sep 6;9:747. doi: 10.3389/fneur.2018.00747 (PMC6135889; doi:10.3389/fneur.2018.00747)
Supplement: Supplementary file 4 [file Table_4.pdf]

SUPPORTING INFORMATION

S4 TABLE. Significant findings of the present study compared to the previous literature published in the Friedreich's Ataxia

	**Present study/Findings**	**Accordance**	**Different**	**Novelty**
**Study design**	Multimodal study: VBM, DTI, fMRI with motor task	*Dogan et al., 2016*: cognitive fMRI study		First three-modal study with fMRI motor-task
**Scanner properties**	3T	*Akhlaghi et al., 2011; Akhlaghi et al., 2012; Georgiou-Karistianis et al., 2012; Bonilha da Silva et al., 2014; Dogan et al., 2016; Harding et al., 2016; Rezende et al., 2016; Selvadurai et al., 2016*	7 T: *Solbach et al., 2014; Stefanescu et al., 2015*	
**Infratentorial regions GM – VBM**	bilateral reduction of GM volume in lobule V, VI, VIII (L>R), the crus of cerebellum, in the vermis posterior lobe, both flocculi, L tonsil in FRDA	*Della Nave et al., 2008b; França et al., 2009; Bonilha da Silva et al., 2014; Dogan et al., 2016; Rezende et al., 2016; Selvadurai et al., 2016*	DN alteration: *Solbach et al., 2014*	GM loss in the lobule VIII, flocculi bilaterally, L tonsil
**Supratentorial regions GM – VBM**	No differences in cerebral GM volume	*Della Nave et al., 2008b; França et al., 2009*	*Loss of GM: Bonilha da Silva et al., 2014; Dogan et al., 2016; Rezende et al., 2016; Selvadurai et al., 2016*	
**DTI WM**	Diffuse FA reduction: deep cerebellar WM, cerebellar peduncles, CST (brainstem/PLIC/subcortical WM close to M1), posterior thalamic radiations and optic radiation, CC, forceps major	*Della Nave et al., 2008b; Della Nave et al., 2011; Rizzo et al., 2011; Clemm von Hohenberg et al., 2013; Vieira Karuta et al., 2015; Dogan et al., 2016; Rezende et al., 2016; Fortuna et al., 2008; Akhlaghi et al., 2014; Mascalchi et al., 2016*		
	No differences of association tracts.		FA reduction in the inferior fronto-occipital fasciculus (*Della Nave et al., 2008b; Vieira Karuta et al., 2015; Dogan et al., 2016*) and in the inferior longitudinal fasciculus (*Della Nave et al., 2008b; Dogan et al., 2016*)	
**DTI metrics correlation to disease severity**	In SCP, forceps, fornix	In cerebellar peduncles, CC: *Della Nave et al., 2008a; Rizzo et al., 2011; Clemm von Hohenberg et al., 2013; Rezende et al., 2016*	No correlations found: *Della Nave et al., 2011; Egger et al., 2014; Vieira Karuta et al., 2015; Mascalchi et al., 2016*	
**fMRI task**	Bimanual task	None	Single handed (domaninant hand): *Mantovan et al., 2006; Akhalaghi et al., 2012; Ginestroni et al., 2012; Stefanescu et al., 2015; Harding et al., 2017*	First study to perform fMRI task with both hands in FRDA

**Intragroup analysis of fMRI**	R-hand task (dominant): activation in L M1, L insula, R superior cerebellar hemisphere (lobules V, VI, VIII). L-hand task: activation of R M1, R insula, L superior cerebellar hemisphere (lobules V, VII, VIII)	*Akhlaghi et al., 2012; Ginestroni et al., 2012; Stefanescu et al., 2015; Harding et al., 2017*		
**Intergroup analysis of fMRI**	Differences only during non-dominant hand task: stronger activation in the L superior cerebellar hemisphere in Hcs. No significant differences were found during the movement of the dominant hand		Reduction of activation in M1, thalamus, DN and lobule V (*Ginestroni et al., 2012; Stefanescu et al., 2015*), increase of activation in parietal cortex, striatum, SMA, lobule VII (*Akhlaghi et al., 2012; Ginestroni et al., 2012; Stefanescu et al., 2015*)	Significant difference during the non-dominant hand motor task, a stronger activation in the L superior cerebellar hemisphere in HCs

Legend: GM: Grey Matter, VBM: Voxel Based Morphometry; DTI: Diffusion tensor Imaging; fMRI: functional Magnetic Resonance Imaging; T: Tesla; L: left, R: right; FA: Fractional Anisotropy, PLIC: posterior limb internal capsule, CST: cortical spinal tracts; WM: White Matter; MD: Medial Diffusivity; CC: corpus callosum; SCP: superior cerebellar peduncles; HCs: healthy controls; DN: dentate nucleus; SMA: supplementary motor area;
